# Alert-Driven Active Defense for IoT-Enabled CBTC Systems Using Bayesian Hypergame Modeling and Hierarchical Reinforcement Learning

**DOI:** 10.3390/s26144475

**Published:** 2026-07-14

**Authors:** Junyi Zhao, Qichang Li, Zhiwei Cao, Zhiyu He, Xiaoyu Zhao, Zhao Sheng, Yong Wang

**Affiliations:** 1 Signal and Communication Research Institute, China Academy of Railway Sciences Corporation Limited, Beijing 100081, China; 19111070@bjtu.edu.cn (J.Z.);; 2 State Key Laboratory of Advanced Rail Autonomous Operation, Beijing Jiaotong University, Beijing 100044, China; 3 From Data to Application Research & Industrization Group, Hangzhou Innovation Institute, Beihang University, Hangzhou 311115, China

**Keywords:** Communication-Based Train Control (CBTC), hypergame theory, APT attacks, Hierarchical Reinforcement Learning (HRL), cyber deception, Bayesian belief updating

## Abstract

Advanced Persistent Threats (APTs) pose a serious threat to Internet of Things (IoT) systems because of their stealthiness, persistence, and ability to adapt to defensive responses. Communication-Based Train Control (CBTC) systems, as IoT-enabled railway signaling infrastructures, have evolved from relatively closed operational environments into interconnected cyber-physical networks, exposing train control systems to coupled cyber intrusion and operational-safety risks. To address this challenge, this paper proposes an alert-driven active defense framework for CBTC systems that integrates Bayesian belief updating, hypergame-based cognitive-bias modeling, and Hierarchical Reinforcement Learning (HRL). The framework converts intrusion detection system (IDS) alerts, network traffic observations, and cyber-physical observations into belief-state, transition, and reward inputs. The Bayesian model estimates attacker type and attack stage, the hypergame model represents deception-induced asymmetric cognition between attackers and defenders, and the HRL decouples strategic defense posture selection from tactical defense execution. The scenario-driven simulations in a CBTC APT defense setting show that the proposed model strategy achieves an 87.1% defense success rate against APT attacks while consuming 62.7% of the normalized defense resources, outperforming DQN, PG, and PPO under the same test conditions. These results suggest that explicitly coupling cyber observations, CBTC operational constraints, and hierarchical deception-aware policies can improve cost-aware active defense for railway signaling infrastructures.

## 1. Introduction

Communication-Based Train Control (CBTC) systems are increasingly evolving from relatively closed signaling environments into interconnected railway cyber-physical systems. This transformation improves operational flexibility and monitoring capability, but it also exposes signaling functions to cyber threats transmitted through open communication links, maintenance terminals, train–ground traffic, and networked control equipment [1,2,3]. In particular, Advanced Persistent Threats (APTs) can penetrate critical signaling assets stealthily, remain active over long periods, and adapt their behavior according to defensive responses. For CBTC systems, such attacks are especially critical because cyber compromise may propagate into operational consequences such as delayed command delivery, abnormal train–ground communication, and unsafe disturbance to train operation. Therefore, CBTC security requires active defense strategies that can mitigate cyber intrusion while respecting safety, latency, and resource constraints in railway operation [4,5,6,7,8,9].

Cyber deception is a representative active defense strategy that uses decoys, traps, and manipulated observations to disrupt the attack chain. Attack–defense interactions are often modeled using game-theoretic frameworks [10,11,12]. However, traditional game theory usually relies on a common-knowledge assumption, under which attacker and defender share a consistent understanding of actions, payoffs, and system states. In practice, this assumption is often too strong. The defender observes only incomplete alerts and traffic evidence, while the attacker may misperceive honeypots, network topology, and defense readiness. Hypergame theory relaxes this common-knowledge assumption by representing each player’s perceived game separately [13]. This makes it suitable for modeling deception-driven cognitive asymmetry. Nevertheless, a hypergame model alone mainly explains asymmetric perception; it does not specify how the defender should update beliefs from noisy CBTC observations or select executable defense actions online.

This limitation becomes more significant in CBTC-oriented APT defense. Although game theory, cyber deception, and reinforcement learning have been studied in cybersecurity, existing studies rarely translate cyber-layer observations into CBTC-specific operational indicators, such as movement-authority (MA) delay, train-interval deviation, and latency-sensitive safety constraints. In addition, reinforcement learning (RL) must explore a large joint space of system states, belief states, and defense actions, which makes training inefficient and obscures the distinction between strategic posture selection and tactical action execution. These considerations motivate an operationally traceable framework in which CBTC observations drive Bayesian belief updating, deception is represented as a controllable cognitive-bias state, and hierarchical learning decomposes defense decision-making into strategic and tactical levels.

To address these challenges, this paper proposes an alert-driven active defense framework that embeds a multi-stage Bayesian-hypergame model into an HRL-based decision process. By leveraging cyber-physical observations from the CBTC system, the framework dynamically updates the defender’s belief regarding the attacker type and attack stage. Meanwhile, it employs the hypergame model to characterize the deception-induced cognitive asymmetry, and utilizes HRL to separate strategic defense selection from concrete defense actions. Through shared observations, state variables, and policy optimization, the framework integrates Bayesian belief updating, hypergame reasoning, deception modeling, and HRL to construct a unified defense loop. The primary contributions of this research are summarized as follows:

- We formulate an APT-oriented active defense for CBTC systems by jointly considering cyber intrusion evidence and railway operational constraints. The proposed formulation explicitly connects IDS alerts, network traffic anomalies, MA delay, train-interval deviation, latency constraints, and resource consumption with defense-state construction and reward design.
- To describe the uncertainty and cognitive asymmetry in CBTC-oriented APT defense, we construct a multi-stage Bayesian-hypergame model. In this model, the defender updates beliefs over attacker types and attack stages from cyber-physical observations, while the attacker’s misperception is characterized by a cognitive-bias factor associated with deception fidelity, honeypot exposure, and attacker feedback.
- We design an HRL-based defense strategy that decouples strategic posture selection from tactical defense execution. This hierarchy reduces invalid action exploration and supports CBTC-specific defense actions under system constraints.
- We conduct scenario-driven experiments in a CBTC APT defense setting to evaluate the proposed framework. The results show that the proposed HRL-based strategy achieves an 87% defense success rate with 63% normalized resource consumption, outperforming DQN, PG, and PPO. Sensitivity analyses on IDS errors, cognitive bias, reward weights, and resource constraints further demonstrate its robustness.

The remainder of this paper is organized as follows. Section 2 reviews related work. Section 3 establishes the multi-stage Bayesian attack–defense game model and attacker type updating. Section 4 details the HRL-based game solving method. Section 5 presents the simulation. Finally, Section 6 concludes the paper and discusses future work.

## 2. Related Work

### 2.1. Implementation Techniques of Cyber Deception

Cyber deception misleads attackers by manipulating the information they observe. Reference [14] proposed a zero-sum one-sided partially observable stochastic game (OS-POSG) model to derive optimal honeypot deployment strategies, which outperforms random policies in limiting compromised IoT devices. To quantify the effectiveness of deception, Sayin et al. [15] developed a cyber-physical system (CPS) deceptive defense framework. Optimal defense strategies are obtained via semidefinite programming to orient adversarial behavior toward the defender’s interests. Wang et al. [16] presented a “Deception-as-Defense” security game framework that leverages deception to actively shape the attacker’s perception of system status. The work transforms the complex optimization of optimal deception strategies into an efficiently solvable finite-dimensional semidefinite program. The literature [17] proposed a distributed denial-of-service (DDoS) Defense Strategy Selection Model that adaptively adjusts defensive measures by attack intensity. It computes Nash equilibria via gradient-based methods to achieve efficient resource utilization and reduced operational overhead. Qin et al. [18] introduced a bio-inspired hybrid active defense framework based on Software-Defined Networking (SDN), which integrates Moving Target Defense and cyber deception. The framework can dynamically reconfigure network topologies and substantially lower the attack success probability. In [19] the authors model dynamic interactions as a weighted two-player graphical game and use a second-order hypergame to capture information asymmetry. The results indicate that proactively leveraging multi-dimensional information asymmetry for quantitative deception significantly improves autonomous systems performance. Cifranic et al. [20] introduced a Decepti-SCADA framework to build an active deception system for resource-limited, real-time operational technology (OT) environments. Kulkarni et al. [21] incorporated hypergames into attack–defense graph models. The authors induced cognitive bias in attackers and proved that defense policies synthesized by exploiting perceptual discrepancies can notably improve the overall defense success rate.

### 2.2. Application of Game Theory

Game theory is a core method in the research of cyber defense strategies. Shen et al. [22] extended the traditional epidemic theory by introducing node dormancy and death states, then established evolutionary dynamics via a differential game model to achieve optimal defense strategy selection for WSN by solving for a saddle-point equilibrium. The follow-up work [23] proposed a two-layer malware propagation-patching model under a hybrid patch distribution mechanism, and derived the optimal defense and patch deployment via optimization theory. Yao et al. [24] proposed a Bayesian–stochastic hybrid game method for cross-layer cyber attacks in CPS, characterizing the evolution process through a stochastic state-transition game and a Bayesian game that captures inherent uncertainty. The optimal defense strategy is solved by the Multi-Agent Bayesian Q-Learning (MABQL) algorithm. Reference [25] modeled the attack–defense process in the Internet of Vehicles as a Stackelberg game model. By optimizing the deployment of security resources in transportation infrastructure, the optimal mixed strategies are obtained for attackers and the Internet of Vehicles defense system, so as to minimize the impact of attacks and improve the detection probability. Jiang et al. [26] developed a Bayesian Stackelberg model for water supply system cyber attacks, and designed DILA and SBHA algorithms to simplify large strategy spaces. Simulations confirm the framework efficiently solves optimal mixed defense strategies under incomplete information, balancing cyber resilience effectiveness and defense costs. Huang et al. [27,28] proposed multi-stage and dynamic Bayesian game frameworks against APT threats to critical infrastructure. By combining Bayesian belief updating and analyzing perfect Bayesian Nash equilibrium, they realized attacker behavior prediction and strategic defense design, validated in the Tennessee–Eastman industrial control process.

### 2.3. Reinforcement Learning in Cybersecurity

Reinforcement learning is widely used in cyber attack–defense. Rouzbahani et al. [29] proposed a multi-layer defense framework integrating snapshot ensemble deep neural network and adaptive deep autoencoder to detect known and unknown threats. Simulation results show 98.82% detection accuracy and 0.97% false positive rate for passive and active attacks. Li et al. [30] proposed an interpretable intelligence-driven APT edge defense mechanism which integrates edge game and explainable artificial intelligence (XAI). The Bayesian-Stackelberg game model introduced with CTI is used to obtain the optimal defense strategy, and DRL is adopted to optimize defense resource allocation. Yao et al. [31] proposed a defense scheme combining Distributed Kalman Filter (DKF) and Hierarchical Deep Q-Network (H-DQN) to address the problem of illegal eavesdropping in wireless networks of the Internet of Vehicles. The DKF estimates node states and H-DQN selects channel strategies, which significantly improves system secrecy and transmission rates in dynamic scenarios. Zhang et al. [32] proposed an intelligence-driven Host Address Mutation (ID-HAM) scheme to solve the problems in active address mutation. The Advantage Actor–Critic (A2C) algorithm enables adaptive learning of attacker scanning behavior, reducing scanning hit rate by up to 25% with negligible communication impact. Liu et al. [33] adopted Partially Observable MDP to analyze attack–defense behaviors and constructed a stochastic game model. The authors employed Deep Recurrent Q-Network (DRQN) to dynamically compute the game equilibrium under bounded rationality and incomplete information. The simulations confirm it effectively improves DRDoS defense performance via fast convergence and online adaptive learning.

In summary, game theory effectively characterizes attack–defense interactions, and reinforcement learning provides feasible solutions for complex threats. However, most existing game models assume attackers and defenders have the same understanding of the game rules, ignoring cognitive bias. Thus, this paper introduces the hypergame model to quantify cognitive differences and enable more rational deceptive defense modeling. A hierarchical architecture decouples the action space, thereby reducing the search difficulty caused by high-dimensional state–action representations and improving the efficiency and quality of the resulting strategies. Table 1 further summarizes the position of the proposed framework relative to representative adjacent paradigms.

## 3. Modeling of the Attack–Defense Game Model for CBTC Systems

### 3.1. The Structure of CBTC

**Figure 1:**
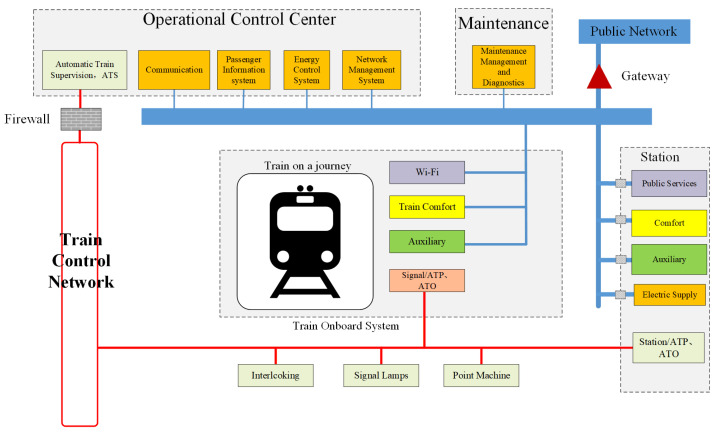
Simplified structure of a communication-based train control (CBTC) system.

In recent years, the operating environment of CBTC systems has gradually evolved from relatively closed signaling networks to highly interconnected railway cyber-physical infrastructures. Figure 1 shows a simplified modern CBTC system adapted from CLC/TS 50701.

The CBTC system includes the operation control center, maintenance center, station system, power monitoring system, wayside equipment, train–ground communication system, and interlocking. These subsystems are geographically distributed and rely on wireless communication, fixed network infrastructure, remote maintenance channels, and distributed wayside devices to support real-time train control and coordination. Such characteristics create attack surfaces at the enterprise boundary, maintenance interface, station server, and communication network. Delayed software updates, constrained maintenance windows, and strict availability requirements may further weaken defense agility and increase the risk of APT penetration into internal railway signaling networks.

### 3.2. CBTC Threat Model

We assume that a capable APT attacker can access the CBTC cyber boundary through enterprise networks, maintenance interfaces, vulnerable station equipment, or compromised edge devices. The attacker can gradually obtain credentials, implant malware, move laterally, and finally disrupt communication or control data. The defender observes IDS alerts, authentication logs, traffic statistics, host events, and cyber-physical operational indicators, but does not directly observe the attacker’s intent or complete internal path. And also the safety-critical constraints require that defensive actions must not violate train separation, MA validity, interlocking logic, or real-time communication requirements.

The attack process is divided into five stages: reconnaissance, initial intrusion, command and control or persistence, lateral movement, and attack impact. In the reconnaissance stage, the attacker scans externally reachable services and maintenance paths. In the initial intrusion stage, the attacker attempts credential misuse, vulnerability exploitation, or malware delivery. In the persistence stage, the attacker hides traces and maintains command channels. In the lateral-movement stage, the attacker expands from IT or maintenance assets toward signaling-related networks. In the attack impact stage, the attacker attempts DoS, replay, tampering, or command-delay attacks that can affect MA updates, communication latency, packet loss, train-interval deviation, or service punctuality.

To make the abstract game states operationally interpretable, Table 2 maps representative attack actions to CBTC subsystems and observable effects. This mapping connects cyber-layer observations, such as IDS alerts and abnormal traffic, with physical-layer safety constraints including MA update latency, train interval, and service punctuality.

The proposed model uses normalized cyber and operational indicators rather than raw alerts directly. At each decision step, the defender constructs an observation vector $o_{k}$

(1) $$o_{k}=\left[z_{k}^{\mathrm{IDS}},z_{k}^{\mathrm{net}},z_{k}^{\mathrm{host}},z_{k}^{\mathrm{MA}},z_{k}^{\mathrm{int}}\right]$$

where $z_{k}^{\mathrm{IDS}}$ denotes IDS alert severity and type, $z_{k}^{\mathrm{net}}$ denotes abnormal traffic intensity, $z_{k}^{\mathrm{host}}$ denotes abnormal login or file activity, $z_{k}^{\mathrm{MA}}$ denotes MA delay, and $z_{k}^{\mathrm{int}}$ denotes train-interval deviation. The mapping between observed CBTC signals, normalized features, model roles, and their operational meanings is summarized in Table 3. These indicators are normalized to [0,1] using engineering thresholds or historical operating ranges, and are then incorporated into the Bayesian likelihood term, the state-transition model, and the cyber-physical reward or cost function.

### 3.3. Multi-Stage Bayesian Attack–Defense Game Formulation

**Figure 2:**
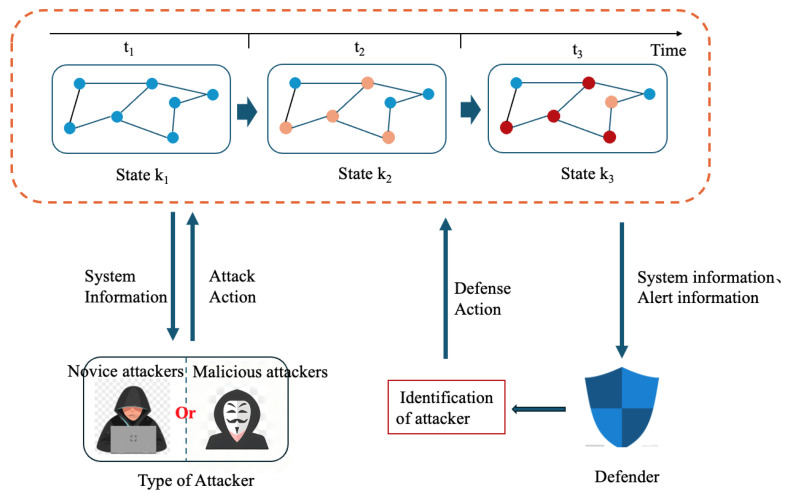
Multi-stage Bayesian game model.

This paper constructs a multi-stage Bayesian game model to characterize the sequential decision-making process of attackers and defenders, as shown in Figure 2. The model follows the five threat stages introduced above. At each stage, both attacker and defender make decisions based on their own observations and beliefs about the other’s type and strategy.

This multi-stage Bayesian game model can be formally defined as a 6-tuple:

(2) ADM-BG=〈N,Θ,S,A,P,U〉

The definitions of each element are as follows:

- $N=(N_{A},N_{D})$ represents the participants in the attack–defense game. $N_{A}$ represents the attacker, $N_{D}$ represents the defender, and |N|=2.
- $\Theta =\{\theta_{1}^{A},\theta_{2}^{A}\}$ represents different types of attackers. $\theta_{1}^{A}$ represents novice attackers which have limited resources and can only perform basic attack methods. $\theta_{2}^{A}$ represents APT attackers which have abundant resources and advanced technical measures. They can pose a significant damage to the system. The binary type space is used in the simulation as a compact proof-of-concept setting. The same Bayesian formulation can be extended to a finer type set, for example ransomware-driven APT, operation-disruption APT, data-theft APT, and supply-chain or zero-day APT, by expanding Θ and defining the corresponding likelihood $\sigma_{A}^{k}\left(a_{A}^{k}|h^{k},\theta_{A}\right)$.
- The Bayesian transition model uses an abstract security-state space:
  $$S=\{s_{0},s_{1},s_{2}\},$$
  where $s_{0}$, $s_{1}$, and $s_{2}$ represent the secure, infiltration, and compromised states. This abstraction captures the coarse security evolution of the CBTC system.
- Action space *A* includes all possible actions of attackers and defenders. Let $A_{A}=\left\{a_{A}^{1},a_{A}^{2},\ldots ,a_{A}^{n}\right\}$ represent the set of attacker actions, such as choosing an attack path, target node, attack method, etc. $A_{D}=\left\{a_{D}^{1},a_{D}^{2},\ldots ,a_{D}^{n}\right\}$ represents the defense measures including deploying firewalls, configuring intrusion detection systems, adjusting system parameters, etc.
- State-transition function *P*:$S\times A_{A}\times A_{D}\to \Delta \left(S\right)$ describes the probability that the system transitions from the current state $s_{k}$ to another state $s_{k+1}$ under the action combination $\left(a_{A},a_{D}\right)$, that is:
  (3) $$P\left(k_{i+1}|k_{i},a_{A},a_{D}\right)=Pr\left(s_{k+1}=k_{i+1}|s_{k}=k_{i},a_{A},a_{D}\right)$$
  Here, $P(k_{i+1}|k_{i},a_{A},a_{D})$ is a conditional probability distribution depending on the action choices of the attacker and the defender. The transition function satisfies the normalization condition:
  (4) $$\sum_{s_{k+1}\in S}P\left(s_{k+1}|s_{k},a_{A},a_{D}\right)=1.$$
- Utility functions $U_{A}$ and $U_{D}$: At each stage, the utility functions of attackers and defenders are represented by
  (5) $$\begin{array}{c} U_{A}:S\times A_{A}\times A_{D}\times \Theta \to R \end{array}$$
  (6) $$\begin{array}{c} U_{D}:S\times A_{A}\times A_{D}\times \Theta \to R \end{array}$$
  The attacker’s utility is related to the degree of successful penetration, resource consumption, and the risk of being detected. The defender’s utility is related to system security, detection effectiveness, defense cost, and the impact on the system.

#### 3.3.1. Attack–Defense Action Space

To capture the asymmetric interaction in the CBTC cyber-physical system, we define the action spaces for both players according to the five APT stages introduced in the threat model. The attacker’s objective for the action space ($A_{A}$) is to penetrate the network layer, maintain access, move toward signaling-related assets, and ultimately disrupt physical train operations. The compact action set $A_{A}=\left\{a_{A}^{r},a_{A}^{i},a_{A}^{p},a_{A}^{l},a_{A}^{m}\right\}$ corresponds to the five representative attack-stage actions used in the simulation, as detailed in Table 4.

The defender’s action space $A_{D}$ is organized into passive, active, and hybrid defense categories. Passive actions focus on monitoring and hardening, such as IDS adjustment, patching, firewall updates, and log inspection. Active actions proactively interfere with the attack process by using deception, redirection, or containment to change the attacker’s perceived environment or restrict compromised assets. Hybrid actions combine alert-driven hardening with selective deception or isolation, aiming to balance defense effectiveness, resource cost, and CBTC operational safety. Table 5 summarizes the representative actions considered by the HRL solver.

#### 3.3.2. Belief Evolution and Behavioral Strategies

In this model, both players adopt behavioral strategies rather than deterministic decisions. For stage *k*, the defender’s behavioral strategy is defined as $\sigma_{D}^{k}:I_{D}^{k}\to \Delta \left(A_{D}^{k}\right)$, where $I_{D}^{k}$ is the defender’s information set at stage *k*. Correspondingly, the attacker’s behavioral strategy at stage *k* is defined as $\sigma_{A}^{k}:I_{A}^{k}\times \Theta \to \Delta \left(A_{A}\right)$, where $I_{A}^{k}$ is the attacker’s information set. The attacker’s choice is conditioned on $\theta_{A}$, which results in divergent attack paths.

Since the defender cannot observe the attacker’s internal decision-making process, it maintains a belief $\mu_{k}^{D}\left(\theta_{A}\right)$, $\theta_{A}\in \Theta$, which represents a probability distribution over the attacker-type set. Let $q_{k}$ denote the current attack stage and $o_{k}$ denote the normalized observation vector defined in Table 3. The alert model assumes that binary alert features, such as probing, exploitation evidence, persistence evidence, and detection events, follow Bernoulli likelihoods conditioned on attacker type and attack stage, while continuous operational features, such as latency or train-interval deviation, are represented by bounded Gaussian likelihoods after normalization. This gives

(7) $$\begin{array}{c} L_{k}\left(\theta_{A}\right)=Pr\left(o_{k}|\theta_{A},q_{k}\right)=\prod_{m\in B}p_{m,\theta_{A},q_{k}}^{b_{m,k}}{\left(1-p_{m,\theta_{A},q_{k}}\right)}^{1-b_{m,k}}\prod_{n\in C}N_{\left[0,1\right]}\left(z_{n,k};{\bar{z}}_{n,\theta_{A},q_{k}},\sigma_{n,\theta_{A},q_{k}}^{2}\right), \end{array}$$

where B and C are the sets of binary and continuous observation features, respectively.

In this study, alert data are constructed by combining platform observations with scenario-driven behavioral profiles. The hardware-in-the-loop (HIL) CBTC platform provides basic observations during simulated attacks, including IDS alerts, traffic anomalies, host events, and operational-state indicators. Because these observations do not cover all attack stages, stealth levels, and IDS false-positive or false-negative conditions, novice and APT attacker behavior profiles are used to supplement sparse alert patterns and initialize the Bayesian observation likelihood. These profiles are treated as scenario-driven likelihood assumptions rather than field-calibrated alert generators. In an operational CBTC deployment, the corresponding likelihood parameters can be further estimated or recalibrated using IDS validation data, traffic baselines, maintenance logs, and safety-operation records. Given the initial prior $\mu_{0}^{D}\left(\theta_{A}\right)$, the defender recursively updates the posterior belief as follows:

(8) $$\mu_{D}^{k+1}\left(\theta_{A}|h^{k+1}\right)=\frac{L_{k}\left(\theta_{A}\right)\sigma_{A}^{k}\left(a_{A}^{k}|h^{k},\theta_{A}\right)\mu_{D}^{k}\left(\theta_{A}|h^{k}\right)}{\sum_{\bar{\theta }\in \Theta }L_{k}\left(\bar{\theta }\right)\sigma_{A}^{k}\left(a_{A}^{k}|h^{k},\bar{\theta }\right)\mu_{D}^{k}\left(\bar{\theta }|h^{k}\right)}.$$

Here, $\sigma_{A}^{k}\left(a_{A}^{k}|h^{k},\theta_{A}\right)$ denotes the probability that an attacker of type $\theta_{A}$ takes action $a_{A}^{k}$ at state $s_{k}$ conditioned on the observed history $h^{k}$. This iterative update allows the defender to dynamically profile the attacker while preserving uncertainty when alerts are noisy or incomplete. Under the assumption of statistical conditional independence between the two players’ decisions, the joint probability of the action profile is expressed as

(9) $$Pr\left(a_{A}^{k},a_{D}^{k}|I_{A}^{k},I_{D}^{k},\theta_{A}\right)=\sigma_{A}^{k}\left(a_{A}^{k}|I_{A}^{k},\theta_{A}\right)\cdot \sigma_{D}^{k}\left(a_{D}^{k}|I_{D}^{k}\right)$$

#### 3.3.3. Cyber-Physical Utility Quantification

In the CBTC cyber-physical system, utility functions quantify the trade-off between operational performance and adversarial interaction. The quantification process integrates cyber-layer disruptions with physical-layer train dynamics to provide a comprehensive metric for decision-making.

1. Cyber-Physical Loss Modeling: The defender’s utility is intrinsically coupled with the system’s ability to maintain train safety and punctual operations under attacks. In this paper, we define the CBTC cyber-physical loss $L_{D}\left(s^{\prime }\right)$ [34] as the core of utility function:
  (10) $$L_{D}\left(s^{\prime }\right)=L_{phys}\left(s^{\prime }\right)+\alpha \Delta F\left(s^{\prime }\right)$$
  The physical layer impact $L_{phys}\left(s^{\prime }\right)$ captures deviations in train motion and scheduling, while the cyber layer impact $\Delta F(s^{\prime })$ quantifies disruptions in the communication network. The coefficient α balances the relative importance of these two components based on the current system phase and operational priorities. Specifically, the physical layer impact is detailed as
  (11) $$L_{phys}\left(s^{\prime }\right)=\sum_{i\in N}\left(\right|V_{t}-V_{target,t}\left|+\beta \right|T_{t}-T_{schedule,t}\left|\right)$$
  $V_{t}$ is the actual speed of train *i*, $V_{target,t}$ is the target speed, $T_{t}$ is the actual arrival time, and $T_{schedule,t}$ is the scheduled arrival time. The coefficient β balance the relative importance of speed and timing deviations.
2. Quantification of Attack and Defense Costs: We define cost functions for both players. The attacker cost $C_{A}$ depends on the attacker type θ and on the complexity of attack measures, including resource expenditure, time investment, and detection risk:
  (12) $$\begin{array}{cc} C_{A}\left(a_{A},\theta \right)= & \lambda_{1}C_{res}\left(a_{A}\right)+\lambda_{2}C_{time}\left(a_{A}\right)\\ \; & +\lambda_{3}C_{risk}\left(a_{A},\mu_{k}^{D}\right) \end{array}$$
  The risk cost $C_{risk}$ is coupled with the defender’s current belief state $\mu_{k}^{D}$. As the defender obtains more information about the attacker type, the risks of detection and eviction increase, which in turn increases the attacker’s cost. The defender cost $C_{D}$ consists of deployment cost, operational overhead, and secondary CBTC service impact:
  (13) $$\begin{array}{cc} C_{D}\left(a_{D}\right)= & \eta_{1}C_{deploy}\left(a_{D}\right)+\eta_{2}C_{overhead}\left(a_{D}\right)\\ \; & +\eta_{3}C_{impact}\left(a_{D}\right), \end{array}$$
  where $C_{deploy}$ represents the effort of enabling a defense action, such as patching, isolation, or honeypot activation. $C_{overhead}$ represents the computational and communication overhead caused by monitoring, redirection, or additional filtering. $C_{impact}$ represents the secondary operational impact on CBTC service, including added communication latency, delayed MA updates, or increased train-interval deviation. The weights $\eta_{1}$, $\eta_{2}$, and $\eta_{3}$ are non-negative and reflect the relative importance of deployment effort, cyber-defense overhead, and operational impact. For reporting and comparison, the defender cost is normalized to a 0–100 scale:
  (14) $$C_{D}^{norm}=100\cdot \frac{C_{D}\left(a_{D}\right)}{C_{D}^{max}},$$
3. Stage and Cumulative Utility Optimization: The defender evaluates the strategy performance from both short-term and long-term perspectives. At each stage *k*, the defender’s stage utility $J_{D}^{k}$ represents the immediate payoff, defined as the expectation with respect to external uncertainties $w_{D}^{k}$:
  (15) $$J_{D}^{k}\left(s_{k},a_{A}^{k},a_{D}^{k},\theta_{A}\right)=E_{w_{D}^{k}}\left[{\bar{J}}_{D}^{k}\left(s_{k},a_{A}^{k},a_{D}^{k},\theta_{A},w_{D}^{k}\right)\right]$$
  It reflects instantaneous trade-off between mitigating current system losses and the resources consumed by high-intensity defense operations. The cumulative utility $U_{0:K}^{D}$ is the total payoff that integrates the evolution of beliefs about the attacker’s type and behavioral strategies of both players:
  (16) $$\begin{array}{cc} U_{0:K}^{D}= & \sum_{k=0}^{K}\sum_{\theta_{A}\in \Theta }\mu_{D}^{k}\left(\theta_{A}|s_{k}\right)\sum_{a_{A},a_{D}}\sigma_{D}^{k}\left(a_{D}|s_{k}\right)\\ \; & \cdot \sigma_{A}^{k}\left(a_{A}|s_{k},\theta_{A}\right)J_{D}^{k}\left(s_{k},a_{A},a_{D},\theta_{A}\right) \end{array}$$
  The defender’s objective is to determine the optimal strategy sequence $\sigma_{D}^{*,0:K}$ that maximizes the cumulative utility:
  (17) $$\sigma_{D}^{*,0:K}=\arg\max_{\sigma_{D}^{0:K}}U_{0:K}^{D}\left(\sigma_{D}^{0:K},\sigma_{A}^{0:K},s_{0},\theta_{A}\right)$$
  By optimizing the long-term payoff, the defender avoids short-sighted decisions and achieves a strategic balance between immediate risk mitigation and the maintenance of long-term operational benefits.

### 3.4. Hypergame Framework for Active Defense

**Figure 3:**
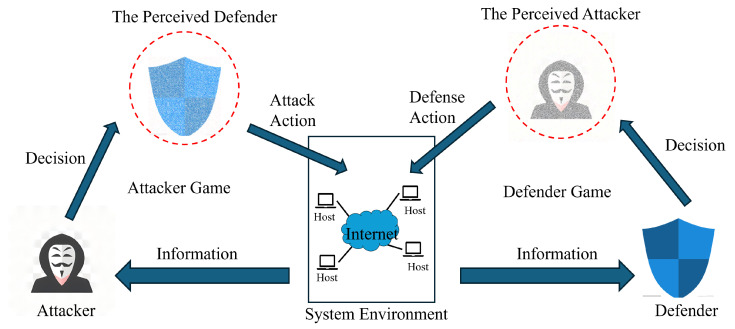
Hypergame model.

Multi-stage Bayesian game models provide a foundation for analyzing attacker–defender interactions. In real APT scenarios, an adversary may misestimate the defender’s available resources, while the defender may underestimate the adversary’s penetration depth. Thus, we introduce a hypergame framework to address cognitive biases in the attack–defense process, as shown in Figure 3.

Formally, given a set of players *N*, each player i∈N perceives a distinct game $G_{i}=\left(N,{\left\{A_{ij}\right\}}_{j\in N},{\left\{U_{ij}\right\}}_{j\in N}\right)$, where $A_{ij}$ denotes the strategy set of player *j* as perceived by player *i*, and $U_{ij}$ denotes the payoff function of player *j* as perceived by player *i*. In cyber defense contexts, we partition players into the attacker $N_{A}$ and defender $N_{D}$, leading to two perceived games:

- The defender’s perceived game
  (18) $$G_{D}=\left(N_{A},N_{D},{\bar{A}}_{A},A_{D},U_{D},{\bar{U}}_{AD}\right)$$
  where ${\bar{A}}_{A}$ and ${\bar{U}}_{AD}$ represent the defender’s perception of the attacker’s strategy set and payoff function.
- The attacker’s perceived game
  (19) $$G_{A}=\left(N_{A},N_{D},{\bar{A}}_{D},A_{A},{\bar{U}}_{DA},U_{A}\right)$$
  where ${\bar{A}}_{D}$ and ${\bar{U}}_{DA}$ represent the attacker’s perception of the defender’s strategy set and payoff function, and $U_{A}$ is the attacker’s true payoff function.

To quantify the attacker’s misperception of the system state *S* and the defender’s strategy set $A_{D}$, we introduce a cognitive bias parameter $\epsilon_{c}\in \left[0,1\right]$. When $\epsilon_{c}=0$, the adversary possesses perfect information. As $\epsilon_{c}$ increases, the adversary’s perception deviates further from the ground truth. In the simulation, $\epsilon_{c}$ is treated as a scenario parameter to analyze the sensitivity of deceptive defense. In practical deployment, it can be interpreted as an estimable variable related to honeypot fidelity, alarm exposure, and attacker observation feedback. For example, high-fidelity decoys and consistent false observations tend to increase $\epsilon_{c}$, whereas honeypot exposure or contradictory observations reduce it. A lightweight online update can be represented as

(20) $$\epsilon_{c,k+1}=\rho \epsilon_{c,k}+\left(1-\rho \right){\hat{\epsilon }}_{c,k},\;0\leq \rho \leq 1,$$

where ${\hat{\epsilon }}_{c,k}$ denotes the bias estimated from current deception evidence and attacker feedback. The cognitive error variables $\delta_{s}$, $\delta_{A}$, and δ are concrete error realizations generated under the bias level $\epsilon_{c}$, rather than additional independent bias parameters. This expression does not change the basic hypergame formulation, but clarifies how the parameter can be calibrated in an operational CBTC defense system.

A practical estimator can be constructed from measurable deception indicators:

(21) $${\hat{\epsilon }}_{c,k}={\mathrm{clip}}_{\left[0,1\right]}\left(\chi_{1}F_{k}^{hp}+\chi_{2}C_{k}^{decoy}-\chi_{3}E_{k}^{exposure}+\chi_{4}B_{k}^{att}\right),$$

where $F_{k}^{hp}$ denotes honeypot fidelity, $C_{k}^{decoy}$ denotes consistency between decoy observations and normal CBTC behavior, $E_{k}^{exposure}$ denotes the risk that the attacker has detected the deception, and $B_{k}^{att}$ denotes attacker feedback, such as repeated interaction with decoys or abnormal persistence near false assets. The coefficients $\chi_{i}$ are deployment-specific calibration weights. In the simulation, $\epsilon_{c}$ is varied over [0,1] to analyze sensitivity rather than estimated from a real railway deployment.

1. State Space Expansion: The adversary’s misperception of the current system state is modeled as
  (22) $$\tilde{s}=s+\delta_{s}$$
  where *s* is the true state, $\tilde{s}$ is the perceived state, and $\delta_{s}$ is the state deviation governed by an error distribution function $F(\epsilon_{c})$. Typically, $F(\epsilon_{c})$ follows a normal distribution where the variance increases with $\epsilon_{c}$. Consequently, the perceived state space is defined as
  (23) $$\tilde{S}=\left\{{\tilde{s}}_{i}|{\tilde{s}}_{i}=s_{i}+\delta_{s_{i}},s_{i}\in S\right\}$$
2. Strategy Space Deviation: The attacker’s misperception of the defender’s strategy set $A_{D}$ is
  (24) $${\tilde{A}}_{D}=A_{D}+\delta_{A}$$
  where $\delta_{A}$ represents the cognitive bias in defensive capabilities. The perceived strategy set is
  (25) $${\tilde{A}}_{D}=\left\{{\tilde{a}}_{D}|{\tilde{a}}_{D}=a_{D}+\delta_{a_{D}},a_{D}\in A_{D}\right\}$$

#### 3.4.1. Design of Active Defense Strategies

Under the hypergame framework, the defender designs active strategies to exploit these cognitive gaps. Deception Strategy: The defender deploys honeypots at critical nodes along the predicted attack path to induce a misperceived state $\tilde{s}$. This forces the adversary to waste resources on high-interaction decoys. The attacker’s utility function is modified as follows:

(26) $$\begin{array}{cc} U_{A}\left(\tilde{s},a_{A}\right)= & \sum_{{\tilde{s}}^{\prime }\in \tilde{S}}P\left({\tilde{s}}^{\prime }|\tilde{s},a_{A},a_{D}\right)[R_{A}\left({\tilde{s}}^{\prime },a_{A}\right)\\ \; & -C_{A}\left(a_{A}\right)]-L_{A}^{\mathrm{honeypot}}\left(\epsilon_{c}\right) \end{array}$$

where $L_{A}^{\mathrm{honeypot}}\left(\epsilon_{c}\right)$ represents the utility loss incurred by attacking decoys, which is proportional to the cognitive bias $\epsilon_{c}$. The probability of the adversary selecting action $a_{A}$ follows a quantal response distribution:

(27) $$\pi_{A}\left(a_{A}|\tilde{s}\right)=\frac{\exp(\beta_{A}U_{A}\left(\tilde{s},a_{A}\right))}{\sum_{a_{A}^{\prime }\in A_{A}}\exp\left(\beta_{A}U_{A}\left(\tilde{s},a_{A}^{\prime }\right)\right)}$$

By optimizing honeypot deployment, the defender maximizes $L_{A}^{\mathrm{honeypot}}\left(\epsilon_{c}\right)$, effectively luring the adversary away from legitimate assets. The defender’s utility is defined by the protection of real resources:

(28) $$U_{D}\left(s,a_{D}\right)=E_{s^{\prime }}\left[R_{D}\left(s^{\prime },a_{D}\right)-L_{R}\left(s^{\prime }\right)-C_{D}\left(a_{D}\right)\right]$$

#### 3.4.2. Hypergame Equilibrium Analysis

Equilibrium analysis in a hypergame identifies stable strategy profiles under asymmetric cognitive conditions and the literature [35] details the proof process of equilibria. In this paper, the equilibrium analysis is used to define the stable target of the defense policy. For a fixed belief $\mu_{D}^{k}$, fixed cognitive-bias level $\epsilon_{c}$, and finite action sets $A_{A}$ and $A_{D}$, each perceived game $G_{A}$ and $G_{D}$ is a finite strategic-form game and therefore admits at least one mixed-strategy equilibrium. The hypergame equilibrium is then interpreted as a pair of mutually stable best responses across the attacker’s perceived game and the defender’s perceived game. When beliefs or $\epsilon_{c}$ change, the perceived games change and the policy must re-equilibrate. Accordingly, the HRL solver reports empirical convergence of policy distributions and rewards rather than a general proof of convergence for a non-stationary hypergame.

- Equilibrium Diversity: Cognitive biases introduce multiple stable solutions as the game structure varies with the attacker’s misperception. Overestimation of target value by attackers can lead to a local equilibrium. Conversely, decreasing cognitive bias $\epsilon_{c}$ shifts the perceived game model and may transition the equilibrium to a different state.
- Local vs. Global Stability: The stability of hypergame equilibria is contingent on the cognitive bias $\epsilon_{c}$. While small $\epsilon_{c}$ allow the game to converge toward traditional Nash equilibria. Large biases $\epsilon_{c}$ result in local equilibria where the adversary is trapped in a suboptimal strategy loop.
- Dynamic Re-Equilibration: Attackers adaptively update their cognitive bias $\epsilon_{c}$ and shift strategy preferences as they collect more information. The defender must dynamically adjust deceptive tactics to maintain a favorable equilibrium.

A hypergame equilibrium (HE) is defined as a strategy profile $\left(a_{A}^{*},a_{D}^{*}\right)$. The attacker’s strategy $a_{A}^{*}$ is optimal given their perceived game $\tilde{s}$, and the defender’s strategy $a_{D}^{*}$ is optimal given their own perceived game *s*. Formally, the attacker chooses the action that maximizes their utility based on their misperceived state:

(29) $$a_{A}^{*}=\arg\max_{a_{A}}U_{A}\left(\tilde{s},a_{A},a_{D}^{*}\right)$$

This means that $a_{A}^{*}$ is the best possible response to the defender’s strategy $a_{D}^{*}$ from the attacker’s perspective. The defender chooses the action that maximizes their utility based on the true system state *s*, knowing the attacker’s strategy $a_{A}^{*}$:

(30) $$a_{D}^{*}=\arg\max_{a_{D}}U_{D}\left(s,a_{A}^{*},a_{D}\right)$$

This ensures that the defender’s strategy is optimal in the real-world context, not just the attacker’s perception. The stability of the HE is defined as follows:

- Attacker’s Optimality: $U_{A}\left(\tilde{s},a_{A}^{*},a_{D}^{*}\right)\geq U_{A}\left(\tilde{s},a_{A},a_{D}^{*}\right)$ for all $a_{A}\in A_{A}$.
- Defender’s Optimality: $U_{D}\left(s,a_{D}^{*},a_{A}^{*}\right)\geq U_{D}\left(s,a_{D},a_{A}^{*}\right)$ for all $a_{D}\in A_{D}$.

When these conditions are met, the attacker will not adjust their strategy $a_{A}^{*}$ and the defender will have no reason to change $a_{D}^{*}$ as they are already maximizing their real-world payoff. This stable profile provides a reference condition under which the defender can protect critical nodes while increasing the attacker’s cost.

## 4. Game Solving via Hierarchical Reinforcement Learning

**Figure 4:**
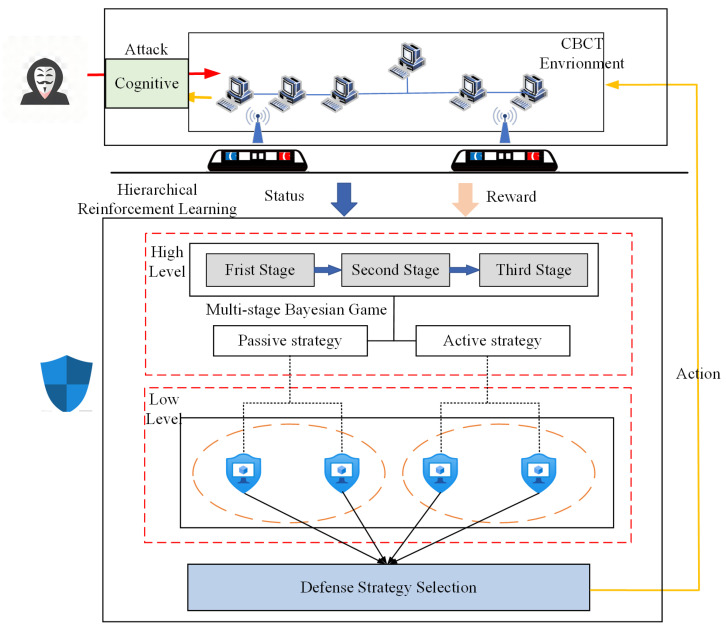
Hierarchical Reinforcement Learning framework.

In this section, we apply a Hierarchical Reinforcement Learning framework. This architecture decouples defense decision-making into two layers: a High-Level Meta-Policy for strategic selection and a Low-Level Sub-Policy for action execution, as shown in Figure 4.

The hierarchical structure reduces the search burden in complex networks by separating strategic intent from tactical implementation. A reinforcement learning agent directly explores the joint decision space formed by system states, belief states, attacker types, and all defensive actions. In contrast, HRL first selects a compact strategic posture σ∈Σ at the high level, and then restricts the low-level search to the defense actions that are meaningful under this posture. Thus, instead of exploring the full space $S\times M\times A_{D}$, the learner handles a smaller high-level problem over $S_{H}\times \Sigma$ and a set of conditional low-level problems over $S_{L}^{\sigma }\times A_{D}^{\sigma }$. Since |Σ| is much smaller than $|A_{D}|$ and $A_{D}^{\sigma }\subseteq A_{D}$, the hierarchy reduces invalid action exploration and improves sample efficiency in the Bayesian-hypergame environment.

The implementation details used in the simulation are summarized in Table 6. The comparison focuses on decision-structure effects under a common scenario-driven simulator rather than on optimizing a specific neural-network architecture. All learners use the same observation variables, attacker scenarios, reward and cost definitions, and episode budget. The DQN, PG, and PPO baselines are treated as non-hierarchical over the same defense-action space, while the proposed HRL solver first selects a defense posture and then searches only the conditional low-level action subset.

DQN, PG, and PPO are implemented as reinforcement learning baseline over the complete defense action space. All compared methods use the same observation inputs, attacker scenarios, reward and cost definitions, stochastic perturbation settings, and training budget. The main difference lies in the decision structure: the baselines directly select actions from the full defense action space, whereas HRL first selects a defense posture and then chooses feasible low-level actions under the corresponding action mask. Each method is evaluated over 10 independent random seeds, and the results are reported with 95% confidence intervals.

### 4.1. High-Level Strategy Selection: Bayesian-Driven Intent Inference

The strategic–tactical partition is predesigned according to CBTC defense knowledge: attacker type inference and defense posture selection are treated as strategic decisions, whereas firewall adjustment, IDS response, isolation, and honeypot deployment are treated as tactical actions. The high-level policy does not have to issue a new strategy at every low-level action step. It can be triggered by a significant posterior-belief update, an attack-stage transition, or a fixed decision period, while the low-level policy updates concrete actions more frequently.

In the meta-policy layer, the defender infers the attacker’s type $\theta_{A}$ and current attack stage using Bayesian updating. This inference shapes the selection of a strategic posture among passive, active, and hybrid defense so that expected cyber-physical loss and defense overhead are jointly minimized.

- State Representation: The high-level state $s_{H}$ includes normalized IDS severity, abnormal traffic intensity, host-event indicators, posterior belief $\mu_{D}\left(\theta_{A}\right)$, attack-stage estimate $q_{k}$, remaining defense-resource budget, MA delay, and train-interval deviation.
- Meta-Policy Q-function: The high-level agent evaluates the expected payoff of choosing a strategy type $\sigma \in \{\sigma_{passive},\sigma_{active},\sigma_{hybrid}\}$. The Q-function is conditioned on the defender’s belief $\mu_{D}\left(\theta_{A}\right)$:
  (31) $$Q^{\left(1\right)}\left(s,\sigma \right)=E\left[U_{D}\left(s,\sigma \right)|\mu_{D}\left(\theta_{A}\right)\right]$$
  where $\mu_{D}\left(\theta_{A}\right)$ represents the posterior belief updated via the Bayesian rule based on observed historical behaviors.
- Strategy Selection: If the inferred attacker type is high-risk and the current stage is impact or lateral movement, the high-level agent prioritizes active or hybrid defense to disrupt the attack. For low-risk types or reconnaissance stages, passive defense is favored to reduce operational overhead. For intermediate-risk states, hybrid defense is selected when selective deception or isolation provides a better utility-cost trade-off than pure active defense.
- Policy Update: The high-level Q-values are updated as
  (32) $$\begin{array}{cc} Q^{\left(1\right)}\left(s,\sigma \right)\leftarrow & Q^{\left(1\right)}\left(s,\sigma \right)+\alpha [R^{\left(1\right)}+\gamma \max_{\sigma^{\prime }}Q^{\left(1\right)}\left(s^{\prime },\sigma^{\prime }\right)\\ \; & -Q^{\left(1\right)}\left(s,\sigma \right)] \end{array}$$
  where $R^{\left(1\right)}$ is the immediate reward for accurate intent inference and effective posture selection, α is the learning rate, and γ is the discount factor for future rewards.

### 4.2. Low-Level Strategy Execution: Hypergame-Driven Deceptive Action

Once the high-level strategy σ is selected, the low-level agent optimizes specific defensive actions by exploiting the attacker’s cognitive error δ induced by $\epsilon_{c}$. This layer translates strategic intent into tactical deception.

- Sub-Policy Q-function: The low-level agent evaluates the effectiveness of a specific action $a_{D}$ against the attacker’s perceived state $\tilde{s}$. The Q-function incorporates the cognitive error parameter δ:
  (33) $$Q^{\left(2\right)}\left(s,a_{D}|\sigma \right)=E\left[U_{D}\left(s,a_{D},a_{A}\right)|\delta \right]$$
  $U_{D}\left(s,a_{D},a_{A}\right)$ is the defender’s utility when executing action $a_{D}$ in response to the attacker’s action $a_{A}$, and δ captures the attacker’s misestimation of the defender’s strategy set.
- Deception Optimization: The Q-function update rule accounts for the impact of δ on state transition probabilities, guiding the sub-policy toward effective deceptive responses:
  (34) $$\begin{array}{cc} Q^{\left(2\right)}\left(s,a_{D}|\sigma \right)\leftarrow & Q^{\left(2\right)}\left(s,a_{D}|\sigma \right)+\alpha [R\left(s,a_{D}\right)\\ \; & +\gamma \max_{a_{D}^{\prime }}Q^{\left(2\right)}\left(s^{\prime },a_{D}^{\prime }|\sigma \right)-Q^{\left(2\right)}\left(s,a_{D}|\sigma \right)] \end{array}$$
  where $R(s,a_{D})$ is the immediate reward for successful deception. By leveraging the hypergame model, the low-level agent maximizes the attacker’s resource exhaustion while minimizing collateral damage to legitimate operations.

### 4.3. Stable Policy Solution and Reward Mechanisms

The HRL framework aims to approach a stable hierarchical policy profile, where the learned high-level strategy and low-level action policy no longer change significantly across training iterations. Because the attack–defense process is simulated as a complex and partially observed environment, the following criteria are used as empirical convergence indicators rather than a formal proof of Nash equilibrium. Stability is achieved through iterative refinement of both the meta-policy and sub-policy.

- Strategy Stability: The learned policy profile is considered stable when the change in strategy distributions π falls below a threshold ξ:
  (35) $$|\pi_{A}^{r+1}-\pi_{A}^{r}\left|<\xi \;\mathrm{and}\;\right|\pi_{D}^{r+1}-\pi_{D}^{r}|<\xi$$
  The threshold ξ is used here to avoid confusion with the discount factor γ. This ensures that the defender’s strategy choices no longer change significantly across iterations.
- Hierarchical Optimality Indicators: The high-level strategy $\sigma^{*}$ is selected when $Q^{\left(1\right)}\left(s,\sigma^{*}\right)=\max_{\sigma }Q^{\left(1\right)}\left(s,\sigma \right)$, meaning no alternative strategy type yields a higher estimated utility. The low-level action $a_{D}^{*}$ is selected when $Q^{\left(2\right)}\left(s,a_{D}^{*}\right)=\max_{a_{D}}Q^{\left(2\right)}\left(s,a_{D}\right)$ under the current strategic posture.

Hierarchical Reward: To guide agents toward a robust equilibrium, we design a tiered reward structure that aligns with both Bayesian inference accuracy and hypergame deception effectiveness:

- High-Level Reward ($R^{\left(1\right)}$): This reward incentivizes accurate attacker profiling and strategic posture selection:
  (36) $$R^{\left(1\right)}=f\left(\mu_{D}\left(\theta_{A}\right)\right)+U_{D}\left(s,\sigma \right)$$
  where f(·) is a function of inference accuracy. Higher rewards are granted when the strategic posture matches the inferred attacker type.
- Low-Level Reward ($R^{\left(2\right)}$): This reward focuses on the efficacy of deceptive execution, quantifying the success of inducing cognitive bias:
  (37) $$R^{\left(2\right)}=g\left(\delta \right)+U_{D}\left(s,a_{D}\right)$$
  where g(δ) quantifies the degree of successfully induced cognitive bias. This ensures that the low-level agent prioritizes actions that steer the attacker toward decoys or isolated network segments.

The two rewards are not optimized as isolated objectives. Instead, they provide level-specific feedback for the same long-term defender utility $U_{0:K}^{D}$. The high-level reward encourages correct attacker profiling and appropriate strategy abstraction, while the low-level reward encourages effective deceptive execution and cost-aware response. Their coordination can be written as a weighted training signal

(38) $$R_{k}=\omega_{1}R_{k}^{\left(1\right)}+\omega_{2}R_{k}^{\left(2\right)}+\omega_{3}U_{D}\left(s_{k},a_{D},a_{A}\right)-\omega_{4}C_{D}\left(a_{D}\right),$$

where $\omega_{i}$ are non-negative weights. This design keeps both layers aligned with the global objective of reducing cyber-physical loss and unnecessary defense overhead. The reward weights are selected after each component is normalized to a comparable range. In the simulation, the default setting emphasizes defense effectiveness while retaining an explicit penalty for resource consumption, and sensitivity runs vary these weights to test whether the learned preference is robust. For operational CBTC deployment, these weights should be calibrated by railway operators according to safety integrity requirements, allowable communication latency, service punctuality targets, and maintenance workload. Actions that violate mandatory train-control constraints are removed from the feasible action set by action masks, rather than being handled only as reward penalties.

In a complex attack environment, defenders must balance high-level posture selection and low-level action execution for different attack threats. By combining Bayesian inference, hypergame modeling, and HRL, the proposed solver implements defense behaviors according to inferred attacker type, attack stage, and cognitive-bias state. The coordination between high-level decision-making and low-level execution improves defense effectiveness while controlling unnecessary system loss.

Algorithm 1 details the training and solving process of the HRL framework.
**Algorithm 1** HRL for Dynamic Defense Strategy Solving**Require:** System state space *S*, belief space M, strategy sets $A_{A},A_{D}$. Learning rates $\alpha_{1},\alpha_{2}$, discount factor γ, convergence threshold ξ, exploration rate $\epsilon_{exp}$.
 1:**Initialize:** 2:$Q^{\left(1\right)}\left(s,\sigma \right)$ and $Q^{\left(2\right)}\left(s,a_{D}\right)$ with random weights; 3:initial belief $\mu_{0}^{D}\left(\theta_{A}\right)$. 4:**for** each episode r=1,2,…,R **do** 5:     Observe initial system state $s_{0}\in S$. 6:     **while** *s* is not terminal **do** 7:          **High-level (Meta-Policy):** 8:          Observe attacker behavior history $h^{k}$. 9:          Update Bayesian belief $\mu_{D}^{k+1}\left(\theta_{A}|h^{k+1}\right)$ using Equation (8).10:         Select strategic posture $\sigma \in \{\sigma_{passive},\sigma_{active},\sigma_{hybrid}\}$ using $\epsilon_{exp}$-greedy policy based on $Q^{\left(1\right)}\left(s,\sigma \right)$.11:         **Low-level (Sub-Policy):**12:         Retrieve attacker cognitive error parameter δ from hypergame model.13:         Execute specific action $a_{D}$ based on    $Q^{\left(2\right)}\left(s,a_{D}|\sigma ,\delta \right)$.14:         **Environment Interaction:**15:         Observe attacker action $a_{A}$, next state $s^{\prime }$, and transitions.16:         Calculate High-level reward:17:         $R^{\left(1\right)}=f\left(\mu_{D}\left(\theta_{A}\right)\right)+U_{D}\left(s,\sigma \right)$18:         Calculate Low-level reward:19:         $R^{\left(2\right)}=g\left(\delta \right)+U_{D}\left(s,a_{D}\right)$20:         **Policy Update:**21:         $Q^{\left(1\right)}\left(s,\sigma \right)\leftarrow \left(1-\alpha_{1}\right)Q^{\left(1\right)}\left(s,\sigma \right)+\alpha_{1}\left[R^{\left(1\right)}+\gamma \max_{\sigma^{\prime }}Q^{\left(1\right)}\left(s^{\prime },\sigma^{\prime }\right)\right]$.22:         $Q^{\left(2\right)}\left(s,a_{D}\right)\leftarrow \left(1-\alpha_{2}\right)Q^{\left(2\right)}\left(s,a_{D}\right)+\alpha_{2}\left[R^{\left(2\right)}+\gamma \max_{a_{D}^{\prime }}Q^{\left(2\right)}\left(s^{\prime },a_{D}^{\prime }\right)\right]$.23:         $s\leftarrow s^{\prime }$.24:     **end while**25:     **Convergence Check:**26:     **if** $∥\pi_{D}^{r+1}-\pi_{D}^{r}∥<\xi$ and $∥\pi_{A}^{r+1}-\pi_{A}^{r}∥<\xi$ **then Terminate:** Stable hierarchical policy profile reached.27:     **end if**28:**end for**


## 5. Results and Analysis

### 5.1. Experimental Setup and Parameterization

**Figure 5:**
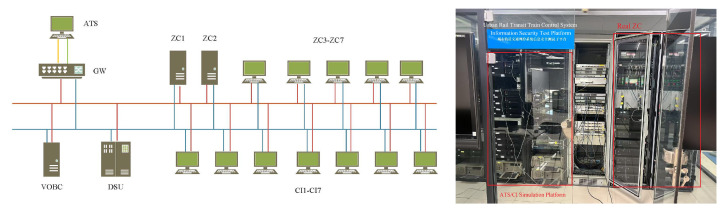
CBTC simulation platform.

Because public CBTC cybersecurity datasets are scarce and real railway signaling systems are safety-critical, real attack experiments cannot be conducted on an operational system. Therefore, this study conducts scenario-driven simulation experiments on a laboratory CBTC test platform. As shown in Figure 5, the CBTC simulation platform includes Automatic Train Supervision (ATS), Zone Controller (ZC), Computer Interlocking (CI), and the Data Communication System (DCS). The platform can reproduce practical CBTC operation scenarios, including route setting, scheduled train departure, and timetable adjustment. In addition, the equipment-interaction information and communication cycles follow CBTC standards.

For simulation purposes, we establish two attack scenarios according to the assumed adversarial capabilities:

- Scenario 1: Novice Attack. This scenario represents attackers with limited resources and basic attack capabilities. The attacker mainly uses straightforward attack methods against exposed CBTC-related components.
- Scenario 2: Advanced Persistent Threat. This scenario represents a sophisticated adversary with stronger persistence, stealth, and adaptability. The attacker can adjust its attack behavior according to system responses and environmental changes.

On this platform, we simulate attack behaviors such as DDoS attacks and MA tampering attacks, and generate seed IDS alerts, traffic anomalies, host events, and CBTC operational-state indicators. Because the platform-generated alert data are still limited in diversity, especially for covering all APT attack stages and stealthy behaviors, this study adopts scenario-driven data augmentation to supplement sparse alert patterns and initialize the Bayesian observation likelihood. Based on this platform, we examine whether the proposed Bayesian-hypergame–HRL decision structure can maintain consistent behavior under predefined attacker profiles, IDS-error assumptions, cognitive-bias levels, and defense-cost settings. The key simulation parameters are summarized in Table 7. The numerical values are not field-calibrated railway parameters; instead, they define a reproducible controlled experimental setting, and their effects are examined through sensitivity analysis. For the likelihood-profile row in Table 7, the tuple order is $\left(p_{probe},p_{exploit},p_{persist},p_{stealth}\right)$.

### 5.2. Simulation Results and Discussion

#### 5.2.1. Intent Inference and Strategy Efficacy

**Figure 6:**
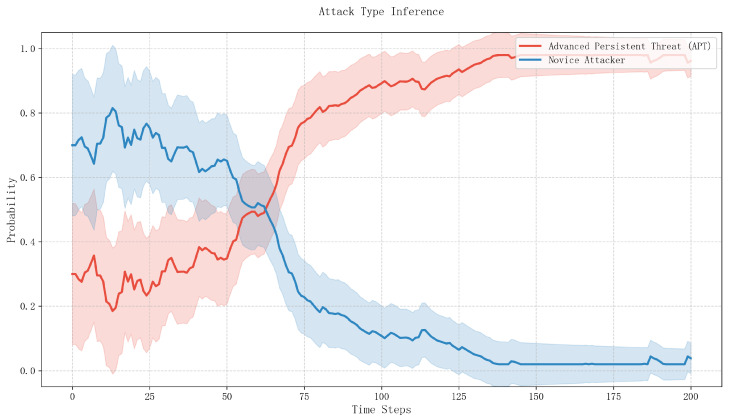
Posterior attacker type inference process based on simulated alert observations.

In the experiment, we assign a prior probability of 0.7 to novice attackers and 0.3 to APT attackers. By continuously acquiring observable alert data, the defender iteratively updates its belief about the adversary’s latent type. With the increase of evidentiary data, the model progressively generates more accurate characterizations of the attacker’s profile. Figure 6 illustrates the convergence of the posterior belief $\mu_{D}$ as alert data accumulates.

**Figure 7:**
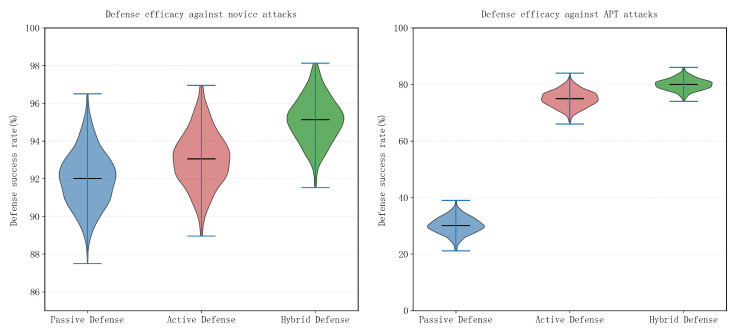
Defense success-rate distributions for passive, active, and hybrid strategies under novice and APT scenarios.

Different defensive strategies demonstrate varying effectiveness against different attackers, as shown in Figure 7. The distributions are generated from 500 stochastic samples per defense mode to visualize variability in the scenario. In scenario 1, passive, active, and hybrid defenses all achieve approximately 90% success. However, in scenario 2, passive defense cannot effectively mitigate attacks, with a defense success rate of only 30%. By contrast, active and hybrid defense strategies both reach approximately 80% success, and their median success rates are higher and more concentrated. These results indicate that deceptive strategies are necessary to enhance system security when facing complex threats.

#### 5.2.2. Strategic Transition and Resource Overhead

**Figure 8:**
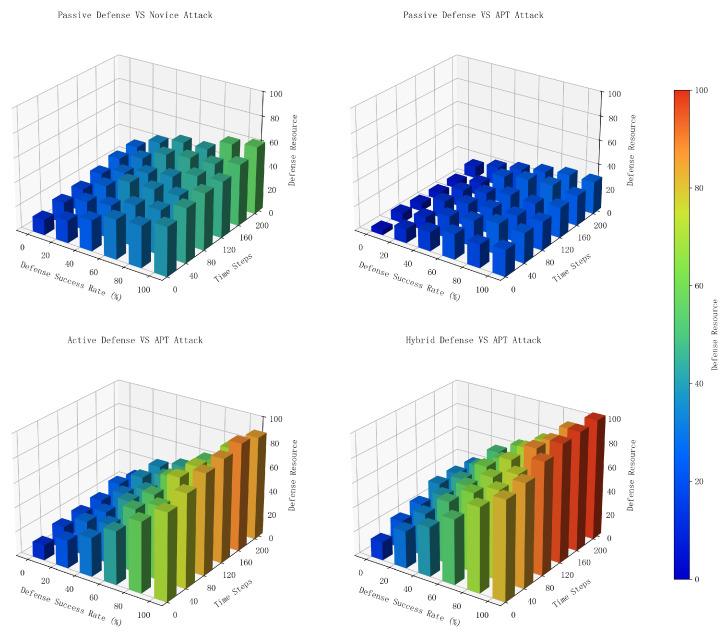
Defense success rate and normalized resource overhead under passive, active, and hybrid defense settings.

Figure 7 shows the defense effectiveness of passive, active, and hybrid strategies against novice and APT attacks, while Figure 8 compares their defense success rate and normalized resource consumption. The resource consumption index follows the normalized defender cost defined in Equation (14). A larger value indicates higher deployment effort, cyber-defense overhead, or secondary operational impact on CBTC service, such as additional communication latency, delayed MA updates, or increased train-interval deviation.

The results show that passive defense is sufficient for Novice attacks, reaching approximately 80% success with a normalized resource consumption of about 45 in the novice-attack scenario. However, it is insufficient against APT attacks, where the success rate remains below 35%. Active defense improves the APT defense success rate to above 90%, but its normalized resource consumption also exceeds 90, which may be undesirable for latency-sensitive CBTC operation. In comparison, hybrid defense achieves approximately 85% success with a lower normalized resource consumption of about 65. This indicates that combining passive monitoring, selective deception, and conditional isolation provides a more balanced trade-off between defense effectiveness and operational overhead.

**Figure 9:**
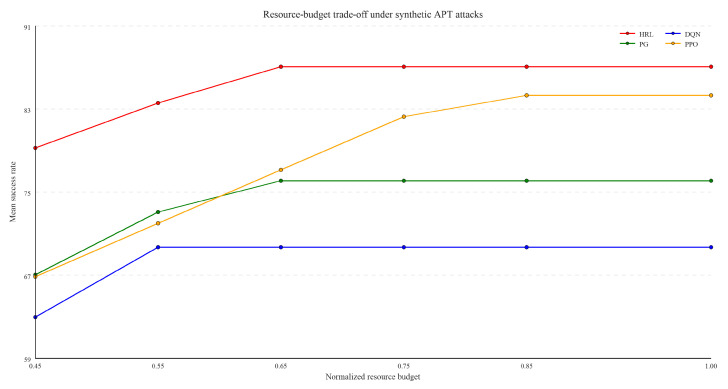
Resource-budget trade-off under scenario-driven APT attacks.

To further clarify the operational meaning of the resource index, an additional resource-budget experiment was conducted over 10 random seeds. The normalized resource budget was varied from 0.45 to 1.00, while the same APT scenario and reward definition were used. Figure 9 shows that HRL improves as the budget increases from 0.45 to approximately 0.65, after which the success rate saturates. Table 8 reports the corresponding CBTC operational-impact indicators for HRL. Increasing the budget improves defense effectiveness, but it also increases MA delay and train-interval deviation because more filtering, redirection, and isolation operations are activated. This result supports using a hybrid policy rather than unrestricted active defense in latency-sensitive CBTC environments.

#### 5.2.3. Impact of Cognitive Bias in Hypergames

In the hypergame model, the attacker’s perception of system state and defense strategies is influenced by cognitive bias $\epsilon_{c}$. In our previous research [36], we predicted the attack path in the CBTC system. Based on the prediction results, we deploy honeypots on the attack path to mislead attacker’s perception. The following results illustrate the impact of cognitive bias on the attacker’s strategy selection and attack utility under various attack scenarios.

**Figure 10:**
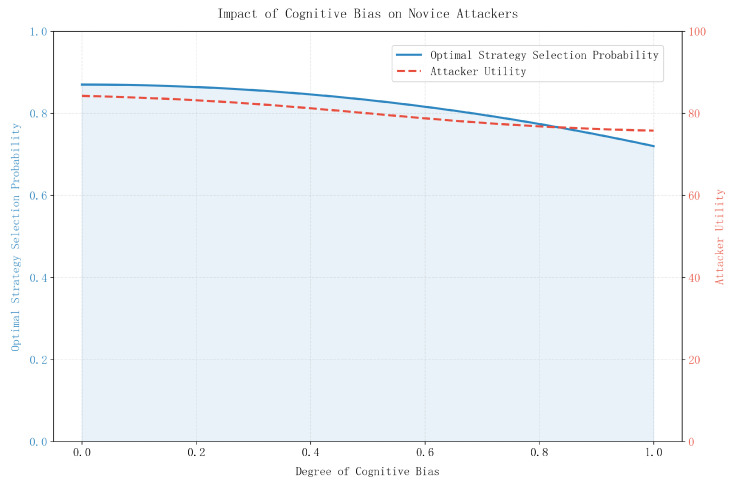
Impact of cognitive bias on novice attackers.

The simulation results in Figure 10 show that cognitive bias has a relatively small effect on the strategy selection and attack utility of novice attackers. This is because novice attackers employ fixed attack strategies and targets, and do not adjust their attack strategies in response to environmental changes.

**Figure 11:**
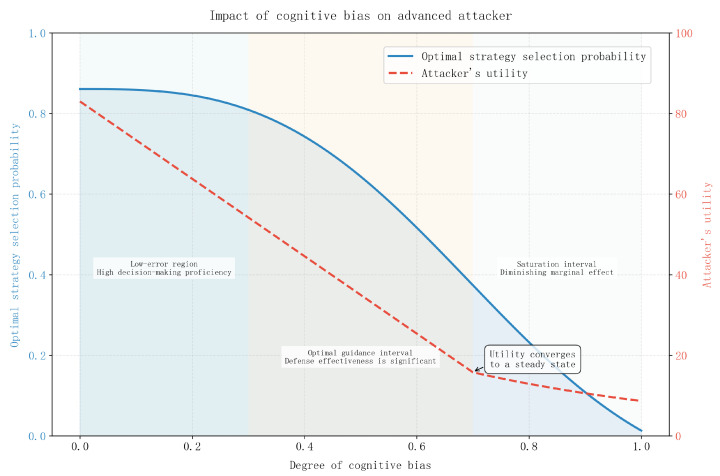
Impact of cognitive bias on APT attackers.

In Figure 11 the probability of APT attackers selecting the optimal strategy and attack utility decreases significantly. When the cognitive bias $\epsilon_{c}<0.3$, the attacker can still select the optimal strategy with a high probability. When the cognitive bias is in the range of $\epsilon_{c}\in \left[0.3,0.7\right]$, there is an obvious deviation between the attacker’s chosen strategy and the optimal strategy, and the attack utility shows a linear downward trend. Once the cognitive bias exceeds 0.7, the probability of selecting the optimal strategy continues to drop, but the attack utility function does not decrease significantly. This suggests that when the cognitive bias $\epsilon_{c}>0.7$, the attacker has already formed a substantial misperception of the system state. Although the utility function still declines with increasing cognitive bias, its marginal effect diminishes, indicating that the effect of the active defense strategy tends to saturate.

The overall simulation results confirm that the hypergame framework effectively captures the impact of cognitive bias on the attacker’s strategy selection and utility. When $\epsilon_{c}\in \left[0.4,0.6\right]$, the active defense strategy is most effective at misdirecting the attacker’s selection and reducing attacker utility. As $\epsilon_{c}$ is increased beyond this range, the strategy’s effectiveness diminishes, while incurring significant additional defensive resource overhead.

#### 5.2.4. Robustness and Sensitivity Analysis

**Figure 12:**
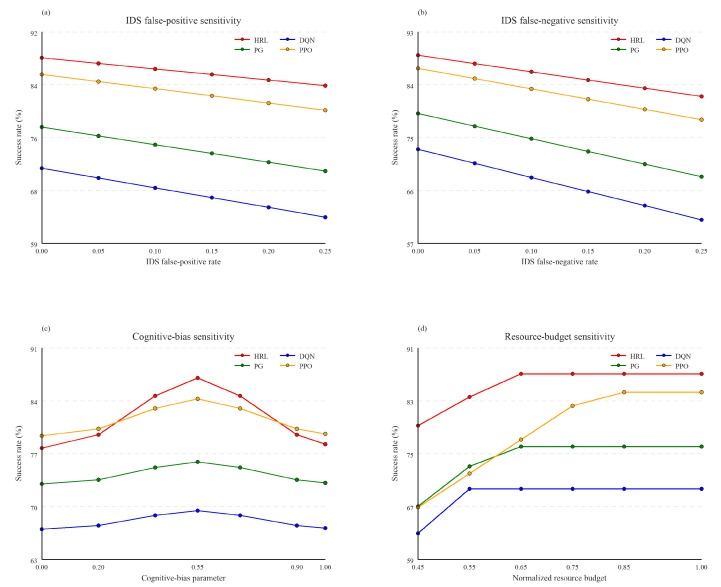
Sensitivity analysis under the APT scenario. Each panel reports mean defense success rate over 10 random seeds for one parameter dimension: (**a**) IDS false-positive rate, (**b**) IDS false-negative rate, (**c**) cognitive-bias parameter, and (**d**) reward resource-penalty weight.

To evaluate the robustness of the proposed framework under different parameter settings, a sensitivity analysis was conducted over four parameters: IDS false-positive rate, IDS false-negative rate, cognitive-bias parameter, and resource-penalty weight in the reward function. Each setting was repeated over 10 independent random seeds. As shown in Figure 12, the performance of all methods decreases as IDS error rates increase. False negatives lead to more significant degradation than false positives because missed attack evidence directly delays Bayesian belief updating. The proposed HRL method shows the most stable performance under both IDS-error settings, as it separates belief-guided high-level defense selection from low-level tactical action execution. The cognitive-bias results indicate that moderate deception strength achieves the highest success rate, while insufficient bias weakens the deception effect and excessive bias produces diminishing returns. The reward-weight analysis further shows that increasing the resource penalty suppresses resource-intensive defense actions and slightly reduces the success rate, which is consistent with the resource-budget trade-off shown in Figure 9.

#### 5.2.5. HRL Convergence and Comparative Performance

In the simulation, HRL is compared with Deep Q-Network (DQN), Policy Gradient (PG), and Proximal Policy Optimization (PPO) over 1000 iterations under the same simulation environment and parameters. DQN, PG, and PPO were implemented as flat solvers over the complete defense action space, whereas HRL adopted the proposed strategic–tactical decomposition with posture-conditioned action masks. Each method was evaluated over 10 independent random seeds, and the results are reported with 95% confidence intervals. Performance was assessed in terms of empirical convergence, defense effectiveness, resource consumption, and operational-impact indicators.

**Figure 13:**
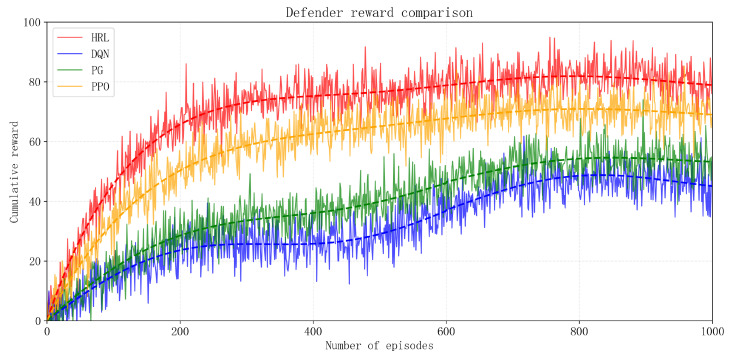
Cumulative reward learning curves for HRL and flat RL baselines in the simulation.

Figure 13 shows the variation trend of cumulative reward during training. Over 1000 iterations, the cumulative reward of HRL increases rapidly within the first 300 iterations and then stabilizes, indicating empirical convergence to a stable hierarchical policy profile in the simulation platform. In contrast, the cumulative rewards of DQN and PG grow slowly at the initial stage, exhibit larger fluctuations in later phases, and do not approach stability until approximately 600 iterations. Although PPO achieves a convergence speed close to that of HRL, its final cumulative reward is lower than that of HRL, suggesting that flat policy optimization is less effective when strategic posture selection and tactical action execution are coupled in one action space.

**Figure 14:**
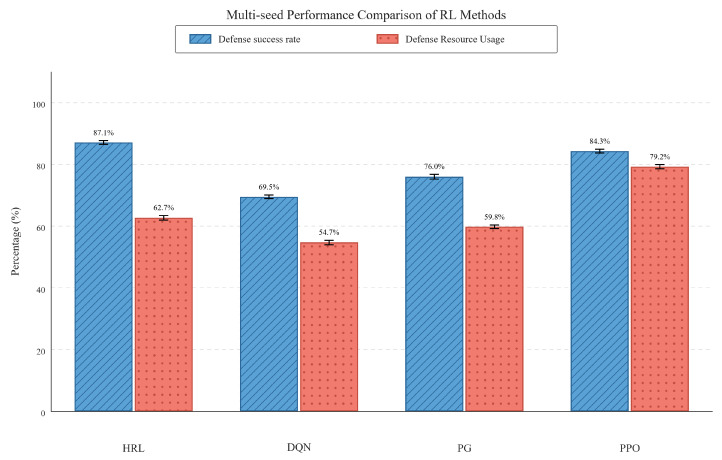
Multi-seed baseline comparison with 95% confidence intervals.

Table 9 and Figure 14 summarize the multi-seed comparison and the corresponding statistical variability; the previous point-estimate comparison figure is omitted to avoid duplicated presentation. Under the APT scenario, HRL achieves a mean success rate of 87.1% with a 95% CI of 86.5–87.7%, compared with 69.5% for DQN, 76.0% for PG, and 84.3% for PPO. HRL consumes 62.7% normalized resources with a 95% CI of 61.9–63.4%, whereas PPO consumes 79.2%. These repeated-run results support the claim that HRL provides the best effectiveness-resource trade-off in the current setting. The results still should not be interpreted as field deployment evidence, because all runs are generated by the scenario-driven environment rather than real CBTC operational data.

To examine whether the performance gain comes from the proposed components rather than only from parameter tuning, a minimal ablation study was conducted over 10 independent random seeds. The ablation is intended to evaluate relative component contribution within the synthetic scenario rather than to provide deployment-level statistics. As shown in Table 10, removing Bayesian belief updating or deception-induced cognitive bias reduces the defense success rate, replacing the hierarchy with a flat RL policy increases resource usage, and removing the resource penalty keeps the success rate similar but increases normalized resource usage.

Combining Figure 13, Table 9 and Figure 14, the collaborative optimization of the high-level and low-level strategy systems in HRL reduces the search complexity and enables faster convergence and superior defense performance. These results demonstrate that in complex attack–defense game scenarios, HRL can not only implement effective defense but also strike a better balance between defense effectiveness and resource consumption.

## 6. Discussion and Conclusions

This study proposes a Bayesian-hypergame–HRL framework for active defense in IoT-enabled CBTC systems. The Bayesian component updates the defender’s belief about attacker type and attack stage from cyber-physical operational indicators. The hypergame component uses the parameter $\epsilon_{c}$ to represent cognitive asymmetry induced by cyber deception, and the HRL component separates strategic defense-posture selection from tactical defense-action execution. The experimental results show that attacker-type inference becomes more stable as alert evidence accumulates. They also show that cyber deception is most effective when $\epsilon_{c}$ falls in a moderate range. In the repeated APT scenario, the proposed HRL strategy achieves an 87.1% mean defense success rate, with a 95% confidence interval from 86.5% to 87.7%, and uses 62.7% normalized defense resources. Under the current test setting, this strategy provides a better balance between defense effectiveness and resource consumption than the non-hierarchical reinforcement-learning baselines.

The results also suggest several practical points for cyber defense in safety-critical CBTC systems. False alerts and missed detections affect belief updating and subsequent action selection, so IDS uncertainty should be modeled explicitly in the likelihood function rather than treated as a deterministic input. Active defense actions must also respect operational constraints. Honeypot redirection, traffic filtering, and host isolation can improve cyber defense, but they should not introduce unacceptable communication latency, MA delay, or disruption to safety-related services. In deployment, deception mechanisms are more appropriate for non-safety-critical mirrored assets, maintenance-side services, or isolated network segments than for the trusted command path itself. Reward weights and resource-cost terms should also be calibrated with operator input so that defense decisions reflect safety integrity, service punctuality, and maintenance workload. These considerations indicate that the proposed framework is better suited as a safety-supervised cyber-defense decision-support module than as an unconstrained autonomous controller.

The applicability of the proposed framework is still limited by the current experimental conditions. Because the study relies on a laboratory CBTC test platform and simulated attack conditions, the obtained alert data are not yet rich enough to cover all attack stages, stealth behaviors, and IDS false-positive or false-negative conditions in real APT campaigns. The reported defense success rates and resource-consumption values should therefore be interpreted as results from a controlled experimental setting. In addition, the current attacker type space is binary, and the model adopts a centralized one-attacker and one-defender formulation. Real APT campaigns may involve more diverse attack objectives, technical paths, and coordinated interactions among multiple parties. The cognitive-bias parameter $\epsilon_{c}$ is also treated as a scenario variable in this study. How to calibrate it online from operational logs, honeypot interaction traces, and attacker feedback remains an open problem.

Future work will improve the realism of the experimental validation. Since the current laboratory platform and simulated attack conditions still differ from real CBTC operation environments, future studies will seek to obtain field security logs and operational records for further validation. Future work will also validate the learned defense policy in more realistic railway operation scenarios and extend the framework to multi-attacker and multi-defender settings with safety-certified action masks.

## Figures and Tables

**Table 1:** Concise comparison with adjacent algorithmic paradigms.

Paradigm	Attacker-Type Uncertainty	Cognitive Bias	Dynamic Defense Learning	Hierarchical Decision-Making	Suitability for CBTC Active Defense
Bayesian game	Yes	No	Limited	No	Medium
Hypergame deception	Limited	Yes	Limited	No	Medium
Flat DRL	No/implicit	No	Yes	No	Medium
Attention/sparse-attention MARL	Partial	No	Yes	Partial	High for distributed settings
Meta-RL	Partial	No	Yes	No	High for fast adaptation
Proposed Bayesian-hypergame + HRL	Yes	Yes	Yes	Yes	High

**Table 2:** Mapping between attack actions and CBTC operational effects.

APT Attack Stage	Target Subsystem/ Access Point	Typical Attack Behavior	Observable Evidence	CBTC Operational Effect
Reconnaissance	Enterprise network, maintenance interface	Network scanning, service probing, topology analysis	Scanning traffic, abnormal connections, IDS alerts	Exposure of system structure and potential entry points
Initial intrusion	Maintenance terminal, station server	Password attack, vulnerability exploitation, malware implantation	Abnormal login, suspicious file transfer, medium-level IDS alerts	Establishment of footholds and local device or traffic anomalies
Command and control/ persistence	Compromised host, maintenance server, edge gateway	C2 communication, backdoor persistence, log hiding	Periodic external connections, missing logs, delayed alerts	Increased stealthiness and long-term persistence risk
Lateral movement	Wayside network, backbone network, network interfaces	Credential theft, privilege escalation, internal scanning	Abnormal traffic, access-pattern changes, high-level IDS alerts	Expanded compromise scope and degraded data transmission
Attack impact	Train–ground communication link, command path	DoS, data replay, traffic tampering, command delay	Increased latency, packet loss, MA delay	Train-interval deviation, service interruption, or degraded operation

**Table 3:** Conversion from CBTC observations to model inputs.

Observed Signal	Normalized Feature	Model Role	CBTC Meaning
IDS alert type and severity	$z_{k}^{\mathrm{IDS}}$	Bayesian likelihood and stage evidence	Alert confidence for different attack stages
Abnormal traffic intensity	$z_{k}^{\mathrm{net}}$	State transition, attacker-action evidence	Scanning, C2 traffic, replay, or DoS pressure
Events log	$z_{k}^{\mathrm{host}}$	Access-level, intrusion evidence	Abnormal login, suspicious file transfer, or privilege escalation
MA update delay	$z_{k}^{\mathrm{MA}}$	Reward penalty, operational-impact term	End-to-end delay of MA update
Train-interval deviation	$z_{k}^{\mathrm{int}}$	Reward penalty, service-regularity constraint	Deviation from the scheduled train interval

**Table 4:** APT attack action set.

Attack Strategy	Primary Objective	Operational Effect
Reconnaissance ($a_{A}^{r}$)	System scanning and profiling	Intelligence acquisition
Initial intrusion ($a_{A}^{i}$)	Credential misuse, vulnerability exploitation, or malware delivery	Initial system access
C2/persistence ($a_{A}^{p}$)	Maintaining command channels and hiding traces	Persistent presence and delayed detection
Lateral movement ($a_{A}^{l}$)	Privilege escalation and internal movement toward signaling-related networks	Expanded compromise scope
Attack impact ($a_{A}^{m}$)	DoS, replay, tampering, or command-delay attack	Physical-layer impact on MA updates, latency, or train interval

**Table 5:** Passive, active, and hybrid defense actions in the CBTC scenario.

Defense Category	Representative Actions	Targeted Subsystem	Main Constraint
Passive defense	IDS tuning, patching, firewall update, log inspection	Station servers, maintenance terminals, network boundary	Low disruption, but limited ability to contain APT lateral movement
Active defense	Honeypot deployment, decoy service, host quarantine, traffic filtering, network segmentation	Maintenance network, edge gateway, compromised host, network segment	Higher resource cost; must preserve safety-command paths and real-time communication
Hybrid defense	IDS-guided patching, selective deception, conditional isolation, adaptive traffic filtering	DCS boundary, train–ground network, critical CBTC interfaces	Balances detection confidence, deception benefit, resource cost, and safety constraints

**Table 6:** HRL implementation.

Item	Setting
State input	Normalized observations, posterior belief, attack stage, resource state, and CBTC operational indicators
High-level policy	Selects defense posture $\sigma \in \{\sigma_{\mathrm{passive}},\sigma_{\mathrm{active}},\sigma_{\mathrm{hybrid}}\}$
Low-level policy	Selects executable defense action $a_{D}\in A_{D}^{\sigma }$ under the chosen posture
Action constraint	Posture-conditioned action mask $A_{D}^{\sigma }$
Learning rule	Q-style hierarchical updates with learning rates $\alpha_{1}$ and $\alpha_{2}$
Exploration strategy	$\epsilon_{\exp}$-greedy action selection
Training budget	1000 training iterations

**Table 7:** Simulation parameters and hyperparameters.

Parameter	Value	Hyperparameter	Value
Learning Rate ($\alpha_{1},\alpha_{2}$)	0.01, 0.005	Discount factor (γ)	0.95
Reward Weights ($\omega_{1},\omega_{2},\omega_{3},\omega_{4}$)	Normalized; varied in sensitivity analysis	Exploration ($\epsilon_{exp}$)	0.1
Cognitive Bias ($\epsilon_{c}$)	[0,1]	Iterations	1000
Seeds	10	Sensitivity levels	5
Network Latency	20–50 ms	Train interval	90 s
Inference Time Steps	200	Stochastic samples for defense-effect distributions	500 per strategy
APT likelihood profile	(0.35, 0.65, 0.75, 0.70)	Novice likelihood profile	(0.65, 0.35, 0.25, 0.30)

**Table 8:** Operational-impact indicators for HRL under different normalized resource budgets.

Budget	Success Rate	Resource Usage	MA Delay (ms)	Interval Deviation (s)
0.45	79.2%	45.0%	90.9	2.53
0.55	83.6%	55.0%	102.7	2.98
0.65	87.1%	62.6%	111.6	3.32
0.75	87.1%	62.7%	111.8	3.33
0.85	87.1%	62.7%	111.8	3.33
1.00	87.1%	62.7%	111.8	3.33

**Table 9:** Multi-seed baseline comparison under the APT scenario.

Method	Success Rate	Resource Usage	Cumulative Reward	Convergence Episode
HRL	87.1 ± 0.6%	62.7 ± 0.7%	79.9 ± 1.0	332 ± 7
DQN	69.5 ± 0.6%	54.7 ± 0.7%	54.9 ± 1.0	662 ± 7
PG	76.0 ± 0.8%	59.8 ± 0.6%	59.5 ± 1.2	601 ± 7
PPO	84.3 ± 0.6%	79.2 ± 0.7%	69.9 ± 0.9	429 ± 8

**Table 10:** Ablation study of the proposed framework.

Variant	Success Rate (%)	Resource Usage (%)	Cumulative Reward
Full model	86.9	63.1	80.3
w/o Bayesian update	79.1	66.1	75.9
w/o cognitive bias	77.6	61.2	75.4
w/o hierarchical structure	84.9	79.6	70.2
w/o resource penalty	86.9	70.6	78.7

## Data Availability

The original contributions presented in this study are included in the article. Further inquiries can be directed to the corresponding author.
